# Comparison of aspiration vs non-aspiration techniques in fine-needle cytology of thyroid lesions

**DOI:** 10.4103/0970-9371.70737

**Published:** 2010-04

**Authors:** Anil Kumar Maurya, Anurag Mehta, N S Mani, V S Nijhawan, Rajeev Batra

**Affiliations:** Department of Pathology, Armed Forces Medical College, Pune, Maharashtra, India

**Keywords:** Thyroid lesions, FNAC, FNNAC

## Abstract

**Aim:**

To compare the efficacy of fine-needle non-aspiration cytology (FNNAC) with that of fine-needle aspiration cytology (FNAC) of thyroid lesions.

**Materials and Methods:**

FNAC and FNNAC techniques were studied in 50 cases of thyroid lesions. All the needle-sampling procedures were done by a single operator. The samples were assessed cytologically and evaluated using five parameters, that is, background blood or clot, amount of cellular material, degree of cellular degeneration, and degree of cellular trauma and retention of appropriate architecture.

**Statistical Analysis:**

Wilcoxon signed rank test was performed using SPSS14 software. Differences between all the individual parameters as observed in FNAC and FNNAC smears were insignificant.

**Results and Conclusion:**

After evaluation of FNAC and FNNAC on the basis of these scores, greater numbers of diagnostically superior samples were obtained by FNNAC; however, by FNAC more number of diagnostically adequate smears were observed. The numbers of unsuitable smears were also more by FNNAC technique.

## Introduction

Fine-needle aspiration cytology (FNAC) for tumors was first described by Martin and Ellis in the year 1930 in the United States. In vascular organs, an alternative method of fine-needle non-aspiration cytology (FNNAC) also known as cytopuncture was developed in France in 1982 by Brifford *et al*.[1] This study was undertaken to compare the efficacy and quality of FNNAC with that of FNAC of thyroid lesions using both techniques in 50 thyroid lesions to ascertain whether it could be chosen as a superior cytodiagnostic procedure in vascular organs, especially the thyroid.

## Materials and Methods

The study population comprised all patients who presented with thyroid swellings at the Department of Pathology (cytology section) from May 2005 to April 2007. After a thorough clinical examination, all the patients were subjected to both FNAC and FNNAC. A total of 50 cases of thyroid lesions were included in this study. The procedure was explained to the patient and verbal consent was obtained prior to performing the procedure. The patients were subjected to FNAC and FNNAC using 23-gauge needles and 10-cc plastic syringes. All the procedures were performed by a single operator. FNAC or FNNAC sampling was carried out randomly with lesions, irrespective of consistency and size of lesions. Every slide was assessed without the prior knowledge of techniques utilized. The study was thus single blind and also prevented the observer bias. The smears were scored according to criteria using a predetermined scoring developed by Mair *et al*.[2] The two sampling techniques were compared using five objective parameters: (1) background blood or clot; (2) amount of cellular material; (3) degree of cellular degeneration; (4) degree of cellular trauma; and (5) retention of appropriate architecture [Table 1].

**Table 1:** Table of point allocation[2]

Criteria	Quantitative description	Point score
Background blood/ clot	Large amount; great compromise of diagnosis	0
	Moderate amount; diagnosis possible	1
	Minimal; diagnosis	2
Amount of cellular material	Minima to absent; diagnosis not possible	0
	Sufficient for cytodiagnosis	1
	Abundant; diagnosis possible	2
Degree of cellular degeneration	Marked; diagnosis impossible	0
	Moderate; diagnosis possible	1
	Minima; diagnosis easy	2
Degree of cellular trauma	Marked; diagnosis impossible;	0
	Moderate; diagnosis possible	1
	Minimal; diagnosis obvious	2
Retention of appropriate architecture	Minimal to absent non-diagnostic	0
	Moderate; some preservation of, e.g., follicle, papillae, acini, etc.	1
	Excellent architectural display closely reflecting histology; diagnosis obvious	2

A cumulative score between 0 and 10 points was obtained for each specimen which was then categorized into one of the following three categories:

Category 1—(Score 0–2) Unsuitable for diagnosis.
Category 2—(Score 3–6) Adequate for cytological diagnosis.
Category 3—(Score 7–10) Diagnosis superior.

### Statistical analysis

The difference in the score for the individual parameter was assessed by Wilcoxon signed rank test using SPSS14 software. All the results were analyzed considering the statistical significance at a level of *P*=0.05.

## Results

The non-aspiration technique yielded less diagnostically adequate but more diagnostically superior smears when compared with aspiration technique. A total of 19 cases were unsuitable for cytodiagnosis by non-aspiration as compared with 17 cases by aspiration technique [Table 2].

**Table 2:** The performance of FNAC and FNNAC technique

Performance	Technique	
	FNAC	FNNAC
Diagnostically superior	20 (40.0)	23 (46.0)
Diagnostically adequate	12 (24.0)	9 (18.0)
Unsuitable for diagnosis	17 (34.0)	19 (38.0)

FNAC, fine-needle aspiration cytology; FNNAC, fine-needle non-aspiration cytology; figures in parentheses are in percentage

*P* value obtained by Wilcoxon signed rank test was not statistically significant in favour of non-aspiration sampling for any parameter. However, the average scores for each parameter favoured non-aspiration sampling than aspiration sampling [Table 3].

**Table 3:** Average score and *P* value for each parameter

Parameter	Aspiration sampling	Nonaspiration sampling	*P val*ue
Background blood or clot	1.16	1.24	>0.05
Amount of cellular material	1.35	1.42	>0.05
Degree of cellular degeneration	1.18	1.32	>0.05
Degree of cellular trauma	1.27	1.29	>0.05
Retention of appropriate architecture	1.13	1.28	>0.05

Non-aspiration sampling displayed more cellular material, less cellular trauma and degenerative changes, better retention of architecture and less likelihood of obscuring by blood. The average score per case was 6.04 by non-aspiration technique and was 5.90 by aspiration technique. Table 4 depicts the frequencies of the various lesions encountered.

**Table 4:** Frequency of various thyroid lesions

Type of lesion	No. of cases	Percentage
Multi-nodular goitre	12	27.27
Thyroiditis	9	20.45
Colloid goitreColloid goitre	8	18.18
Colloid cyst	5	11.33
Follicular lesion	5	11.33
Follicular neoplasm	3	6.81
Toxic goitre	1	2.27
Papillary carcinoma	1	2.27

Total	44	100

## Discussion

FNAC, since its inception in 1847, has passed through two phases of initial scepticism and interim enthusiasm and has successfully reached the final stage of acceptance as identified by Orell[3] in his analysis of steps by which the assessment of innovative diagnostic practice progresses. FNAC is widely accepted as the primary method for diagnosis of thyroid lesions. The cytologist faced the common problem in interpreting the hemorrhagic material from thyroid obtained by FNAC.[4] To overcome this inherent problem, an alternative technique FNNAC also called cytopuncture or fine-needle capillary sampling was used first in France for breast tumor and later for orbital and periorbital tumors. The thyroid gland, which is very vascular, often yields aspirate markedly admixed with blood. It has been suggested that FNNAC sampling, by eliminating the negative suction pressure employed in FNAC, decreases the dilution of thyroid cells by blood and the scientific basis was explained by Santos and Leiman.[4] Methods such as suction of material with a needle bore rely on the property of capillary tension in the narrow channel. The fluid or semi-fluid ascends into the narrow tube in inverse proportion to the diameter of that tube or capillary.[5]

The important advantage of FNNAC sampling is easy operation and absolute control over operating hand, especially for neck, breast, cutaneous or subcutaneous tissue.[5] The FNNAC also allows a better perception of tumor consistency.

**Figure 1:**
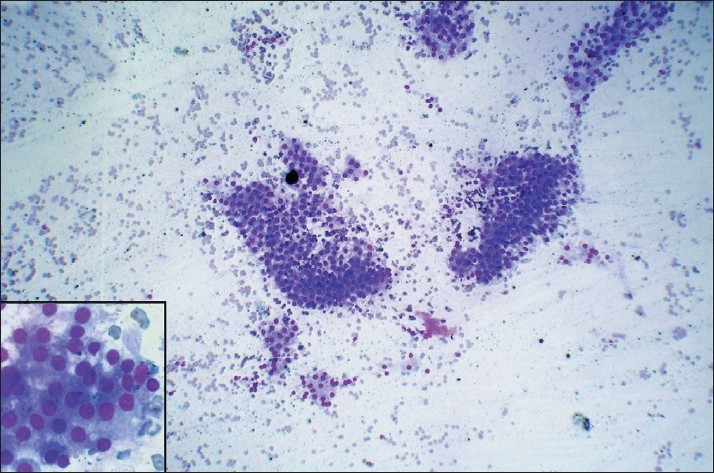
FNNAC smear of colloid goitre showing less blood in the background in comparison to FNAC smear [Figure 2] (Leishman-Giemsa, ×200)

**Figure 2:**
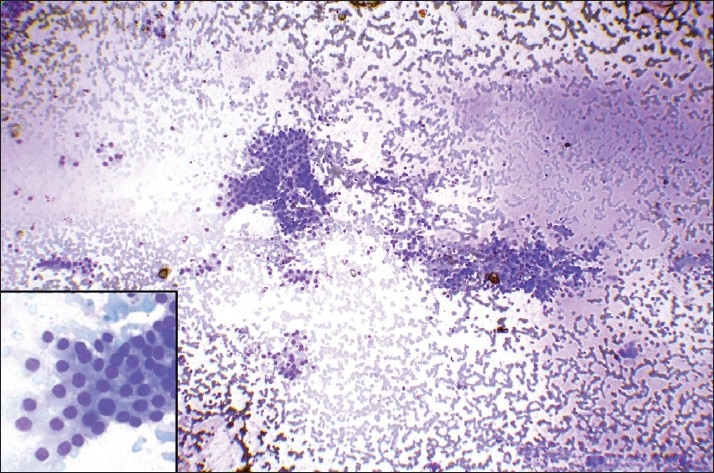
FNAC smear of colloid goitre showing more blood in the background in comparison to FNNAC smear [Figure 1] (Leishman-Giemsa, ×200)

**Figure 3:**
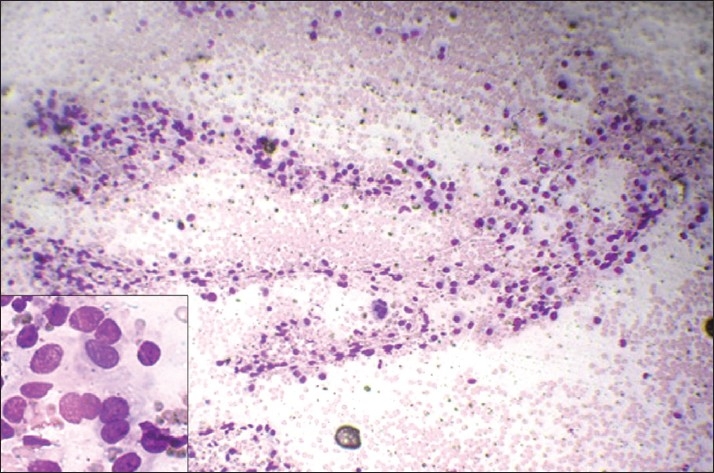
FNAC smears of follicular neoplasm showing low cellularity and more trauma in comparison to FNNAC smear [Figure 4] (Leishman-Giemsa, ×200)

**Figure 4:**
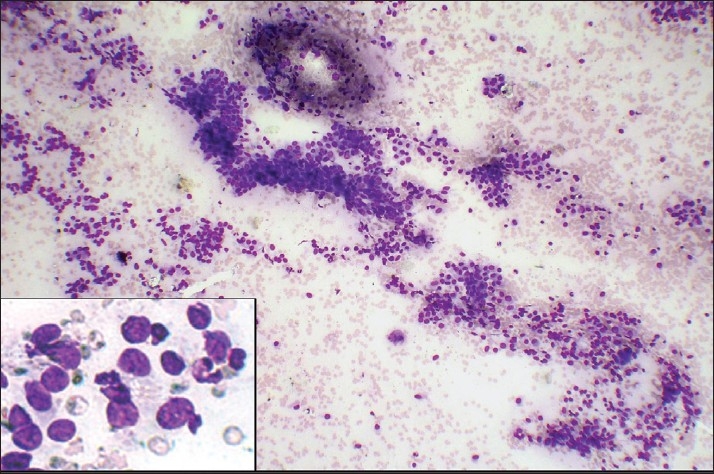
FNNAC smear of follicular neoplasm showing hypercellularity, less trauma and better retained architecture in comparison to FNAC smear [Figure 3] (Leishman-Giemsa, ×200)

Results when compared for background blood contamination supported the non-aspiration technique [Figures 1 and 2] but were not statistically significant. Similar to the study by Meherbano *et al*.,[6] the amount of cellular yield was found to be better by non-aspiration [Figures 3 and 4], but the difference was not statistically significant similar to study by Mair *et al*.[2] However, Jayaram and Gupta[7] observed that cellularity was higher in aspiration smears than in non-aspiration smears in most of the goitres. Cellular degeneration [Table 3] was greater in aspiration similar to the study done by Ghosh *et al*,[8] but the difference was not statistically significant. The degree of cellular trauma was less in non-aspiration as compared with aspiration [Figures 3 and 4] similar to the study done by Ghosh *et al*.[8] but the difference was not statistically significant. Non-aspiration smears yielded better retention of architecture [Figures 3 and 4] with similar findings reported by others.

For the five parameters studied objectively, there was no statistically significant difference observed between the two techniques, and a similar result has been found by Haddadi-Nezhad *et al*.[9] Whereas others observed a statistically significant difference in the total score in favor of non-aspiration as compared with aspiration technique, FNNAC producing a better quality of specimen.[10,11]

In the present study, more diagnostically superior and less diagnostically adequate samples were obtained more by non-aspiration technique in comparison to aspiration technique. It was observed that the percentage of inadequate sampling was more with non-aspiration (38%) than with aspiration (34%) technique in contrast to the observations of Santos and Leiman[4] and Ciatto *et al*.[12] With the utilization of both the techniques for each case, the inadequacy was reduced to 12% as compared with 9.5% observed by Kamal *et al*.[6]

## Conclusions

Both the techniques have their own merits and demerits and neither is superior to the other. By combining both the techniques, better diagnostic accuracy can be achieved. However, FNNAC technique is easier to perform with better patient compliance.

## References

[1] Zajdela A, Zillhardt, Voillemet N (1987). Cytological diagnosis by fine needle sampling without aspiration. Cancer.

[2] Mair S, Dunbar F, Becker PJ, Du Plessis W (1989). Fine needle cytology: Is aspiration suction? A study of 100 masses in various sites. Acta Cytol.

[3] Orell SR (1982). Fine needle aspiration in perspective. Pathology.

[4] Santos JEC, Leiman G (1988). Non aspiration fine needle cytology: Application of a new technique to nodular thyroid diseases. Acta Cytol.

[5] Papanicolaou GN, Traut HF (1943). Diagnosis of uterine cancer by the vagina smear.

[6] Meherbano MK, Arjune DG, Kulkarni HR (200). Comparative study of fine needle aspiration and fine needle capillary sampling of thyroid lesions. Acta Cytol.

[7] Jayaram G, Gupta B (1991). Non aspiration fine needle cytology in diffuse and nodular thyroid lesion: A study of 220 cases. .Acta Cytol.

[8] Ghosh A, Mishra RK, Sharma SP, Singh HN, Chaturvedi AK (2000). Aspiration vs non-aspiration technique of cytodiagnosis –A Critical evaluation in 160 cases. Indian J Pathol Microbiol.

[9] Haddadi-Nezhad S, Larijani B, Tavangar SM, Nouraei SM (2003). Comparative of fine non-needle aspiration with fine needle aspiration technique in cytological studies of thyroid nodule. .Endocr Pathol.

[10] Dey P, Shashirekha, Ray R (1994). Fine needle sampling without suction in intra-abdominal lesion. Acta Cyta.

[11] Romitelli F, Di Stasio E, Santoro C, Iozzino M, Orsini A, Cesareo R (2009). A comparative study of fine needle aspiration and fine needle non- aspiration biopsy on suspected thyroid nodules. Endocr Pathol.

[12] Ciatto S, Lossa A, Cicchi P (1989). Non aspiration fine needle cytology of thyroid tumors’. Acta Cytol.

